# Comparison of Three Methods of Umbilical Cord Management in Late Preterm and Term Newborns on Hemoglobin and Ferritin Levels at Six Weeks of Age: A Randomized Controlled Trial

**DOI:** 10.7759/cureus.59046

**Published:** 2024-04-26

**Authors:** Brajendra Singh, Rakesh Kumar, Saikat Patra, Neetika Bansal, Gaurav Singh, Kasi Raghava, Santosh K Lodhi, Amit Panchal, Surendra Kumar, Ruchi Verma

**Affiliations:** 1 Neonatology, Himalayan Institute of Medical Sciences, Dehradun, IND; 2 Pediatrics, Maharaja Agrasen Medical College, Agroha, Hisar, IND; 3 Obstetrics and Gynaecology, Government Institute of Medical Sciences (GIMS), Greater Noida, IND

**Keywords:** newborn, serum ferritin, hemoglobin, umbilical cord milking, delayed cord clamping

## Abstract

Background: Umbilical cord milking (UCM) and delayed cord clamping (DCC) are strategies that improve the hemodynamic condition of the newborn and also increase the storage of iron. This study aimed to compare the effects of DCC with or without milking in late preterm and term neonates at different time intervals after birth (60, 120, and 180 seconds) on hematological and hemodynamic parameters in neonates at six weeks of age.

Materials and methods: In this double-arm, parallel-group, triple-blind, and active-controlled trial, all 150 eligible neonates were randomized with allocation concealment into three groups: Group A (DCC with UCM at 60 seconds), Group B (DCC with UCM at 120 seconds), and Group C (only DCC for 180 seconds). Hemodynamic parameters were recorded and compared during the first 48 hours, and hematological parameters were compared at six weeks of age.

Results: At six weeks, a significant difference in hemoglobin levels was noted between Groups A, B, and C (p<0.001). The difference in serum ferritin values at six weeks was also statistically significant in comparisons across all three groups (p=0.003). Regarding secondary outcomes examined, hemodynamic parameters and the incidence of neonatal hyperbilirubinemia were found to be comparable at 48 hours after birth.

Conclusion: DCC followed by UCM at 120 seconds and DCC till 180 seconds proves superior to DCC with UCM at 60 seconds in preserving elevated hemoglobin levels and iron stores in neonates at six weeks of age. DCC for 180 seconds yielded comparable results, followed by UCM at 120 seconds. All three methods are considered safe and effective without compromising the neonate's hemodynamics.

## Introduction

Anemia due to iron deficiency is a major health issue in infants born in developing countries [1]. According to the fourth survey of the Indian National Family Health Survey (NFHS-4) conducted in 2015, around 60% of infants of age group 6-11 months were found to be anemic, while it was 70% in Haryana, India (the site of our study) [2]. Both delayed cord clamping (DCC) and umbilical cord milking (UCM) are associated with increased iron stores in the newborn [3], but there is a difference in the dynamics of cerebral blood flow in each strategy [4]. The DCC after 30-180 seconds of birth enhances the blood transfusion from the placenta to neonate, which further increases the hematological parameters and iron stores in both preterm and term neonates. The amount of blood received from the placenta via placental transfusion after birth is approximately 80 mL by one minute and 100 mL by three minutes [5]. This further improves cerebral oxygenation, prevents iron deficiency anemia in infancy, and decreases the need for blood transfusion [6]. It also increases the level of stem cells in newborns from the mother without any abnormal effect [7]. There are many trials and studies to assess the changes in the value of hematological parameters after DCC and early cord clamping versus DCC, but there is no study that has compared the impact of DCC only versus DCC with UCM at a different time after delivery and its effect on hemoglobin and iron status [1].

According to guidelines suggested by the American Academy of Pediatrics (AAP), DCC should be practiced routinely in all deliveries, except in those where babies require resuscitation or are non-vigorous at birth [8]. As per systematic reviews, DCC may not be completely safe. Adverse effects of DCC on maternal and neonatal outcomes are post-partum hemorrhage, the need for phototherapy, polycythemia, and neonatal hyperbilirubinemia (NNH) [9]. However, the quality of evidence was low in the above systematic review study, probably because of variations in study methodologies. Most studies were restricted to comparing ECC with DCC alone; of the very few studies that evaluated different time intervals for performing DCC [10], the timing of DCC remained variable. Various scientific bodies, including the AAP [11], National Institute for Health and Care Excellence (NICE) [12], and World Health Organization [13], endorse DCC, but there is no similarity in its definition.

The timing of DCC proposed by the AAP varies from 30 seconds to five minutes or until the cessation of pulsation in the umbilical cord. Thus, there exists a wide variation in the various studies on DCC and its clinical execution [14]. DCC for three minutes is ideal, but for practical purposes, a minimum duration of one minute is recommended. In a busy setting, the combination of DCC with UCM may be beneficial in term and late preterm neonates to achieve optimal hematological benefits.

This study aimed to compare the effects of DCC and UCM at various time intervals in the different allocated groups on hematological parameters in infants at birth and at six weeks of age. The primary objective of this study was to compare the effects of DCC with or without milking in late preterm and term neonates at different time intervals after birth (60, 120, and 180 seconds) on hematological parameters (hemoglobin and ferritin levels) in neonates at six weeks of age. The secondary objectives of this study were to study the hemodynamic parameters (heart rate (HR), respiratory rate (RR), and blood pressure (BP)) and side effects of placental transfusion (respiratory distress, polycythemia, NNH, and jitteriness) in the three groups.

## Materials and methods

This was a randomized, triple-arm, parallel-group, triple-blind, active-controlled trial conducted in the Department of Pediatrics at a tertiary-level care center in a rural area of Haryana. The study was carried out from September 2019 to August 2020 after receiving approval from the Institutional Ethics Committee for Human Research. Prior to participant enrollment, the study was registered with the Clinical Trial Registry of India (CTRI/2020/05/025318).

A sample size of 47 patients per group was initially determined to be necessary to detect statistical significance with an effect size of 0.20 at an alpha of 0.05 and a power of 80%. However, we enrolled 50 cases per group to accommodate potential post-randomization attrition. A total of 150 neonates were enrolled and randomized in our equivalence trial. Randomization of participants into the three groups was performed using a computer-generated random number table with a block size of 9. Allocation concealment was maintained using the sequentially numbered opaque sealed envelopes (SNOSE) method. Each envelope contained a slip indicating the group and the respective method of cord clamping, which was revealed to the attending pediatrician and gynecologist upon opening by the duty staff.

Triple blinding was ensured at the level of participants, investigators, and analysts. The investigator remained blinded to group allocation as the intervention was carried out by trained pediatricians and gynecologists according to the instructions in the sealed envelope. Following obtaining written informed consent, the duty staff opened the sealed envelope in the presence of the pediatrician and gynecologist. The envelope was retained by the center's nurse in charge for future reference. The investigator completed the case study proforma based on the envelope's serial number, and the group assignment was recorded on the form after the study sample size was reached. The anonymized data for all three groups were sent to a statistician for analysis.

Late preterm (≥34 weeks) and term neonates born via normal delivery or cesarean section, with a birth weight of ≥2 kg and who cried immediately after birth, were eligible for enrollment after parental written informed consent was obtained. Exclusion criteria included multiple births (twins or triplets), neonates with umbilical cord length <25 cm, those delivered by instrumental delivery (forceps or vacuum), non-vigorous newborns at birth, those with perinatal asphyxia, ABO and Rh incompatibility, those who later developed respiratory distress or sepsis requiring NICU admission, babies whose parents refused consent for blood sampling, and babies who received iron supplements in the first six weeks of life. Obstetricians, obstetrics residents, and pediatric resident doctors involved in delivery and neonatal resuscitation underwent training in cord milking techniques through video demonstrations and live sessions conducted by the principal investigator.

Following standard protocols, all newborns received routine care, including early initiation of breastfeeding. For the study, neonates were assessed by the investigator at birth, 24 hours, and 48 hours of life for feeding, respiratory symptoms, signs of sepsis, and hemodynamic parameters. HR, RR, and BP were recorded at different intervals. Blood pressure was measured using non-invasive oscillation in the right arm. Neonates were also monitored for the side effects of placental transfusion.

**Figure 1:**
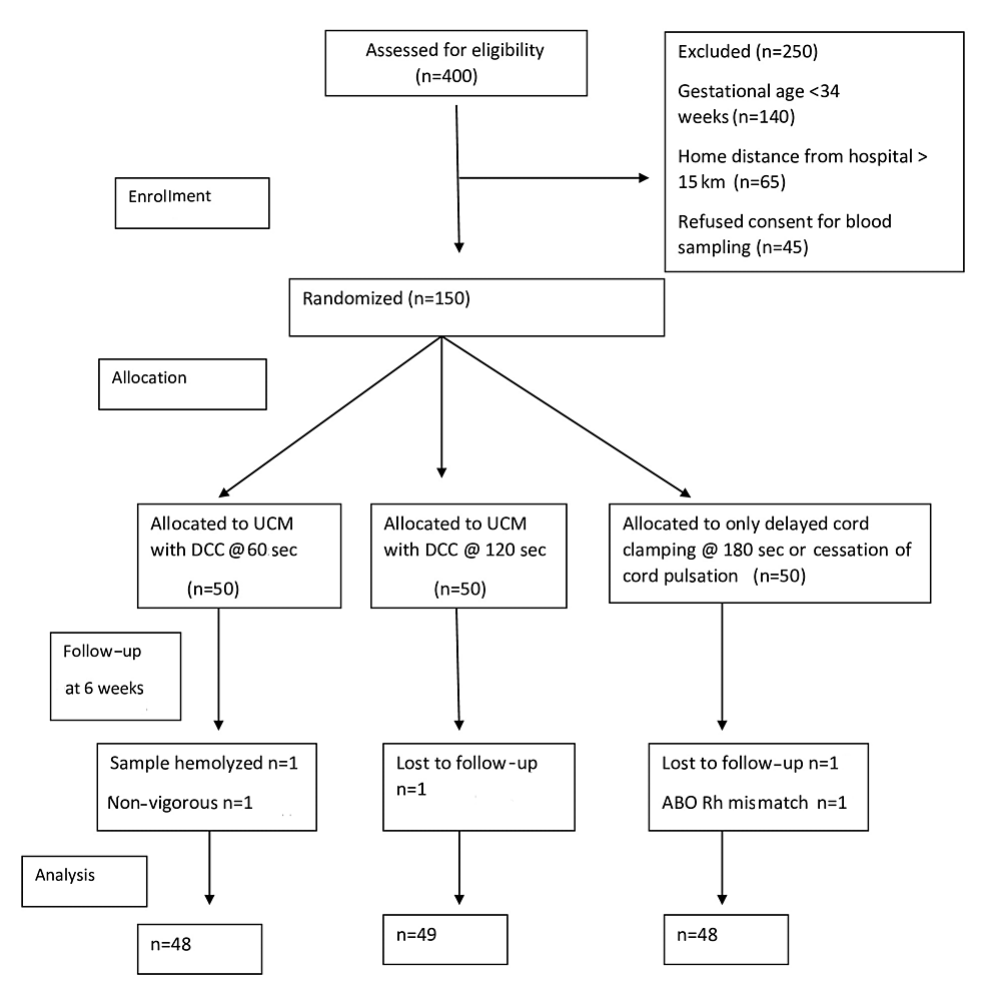
Consort flow diagram of the study.

Cord blood samples were collected at birth for hemoglobin and packed cell volume (PCV) measurements. Samples were again collected for hemoglobin and serum ferritin assessment at six weeks of age. Contact details of the parents were collected for follow-up, and weekly inquiries were made about the newborns' well-being. Follow-up at six weeks of age was ensured for further assessments aligned with the immunization schedule. The CONSORT flow diagram depicting the study plan is presented in Figure 1.

Statistical analysis was performed using Microsoft Excel and IBM SPSS Statistics for Windows, Version 20.0 (Released 2011; IBM Corp., Armonk, New York, United States). Descriptive statistics were used to describe the data, presented as mean±SD (or median) for quantitative data and frequencies (%) for qualitative (categorical) data. A comparison of categorical variables was conducted using ANOVA, t-tests, and two-proportion Z-tests as appropriate. A p-value of <0.05 was considered statistically significant.

## Results

There was a similarity in the three groups in terms of gender, mode of delivery, gestational age, birth weight, and APGAR (Appearance, Pulse, Grimace, Activity, and Respiration) scores (Table 1).

**Table 1:** Baseline variables of all three groups. APGAR: Appearance, Pulse, Grimace, Activity, and Respiration; DCC: delayed cord clamping; UCM: umbilical cord milking.

Baseline variables	Group A (DCC with UCM at 60 seconds) (N=48) (mean±SD)	Group B (DCC with UCM at 120 seconds) (N=49) (mean±SD)	Group C (only DCC for 180 seconds) (N=48) (mean±SD)	p-value
Late preterm (n)	6	5	6	0.92
Term (n)	42	44	42	
Gestational age in weeks (mean±SD)	38.70±1.98	38.86±1.67	38.38±1.69	0.39
Birth weight (mean±SD) in kg	3.02±0.515	2.92±0.488	2.84±0.510	0.20
APGAR score (mean±SD)	8.46±0.542	8.50±0.544	8.46±0.542	0.91

**Figure 2:**
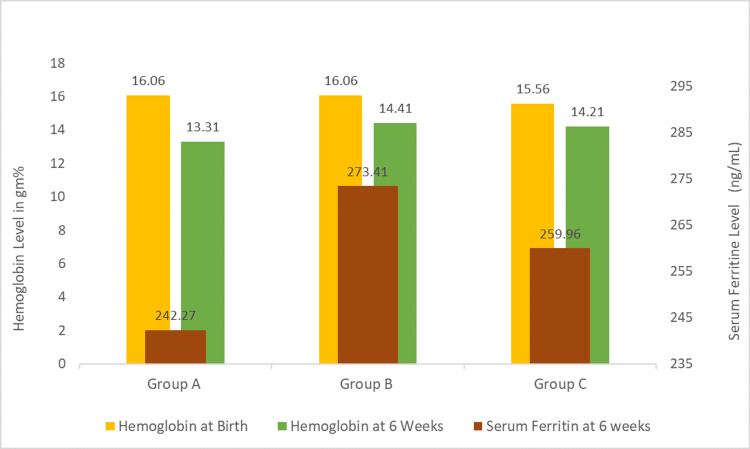
Comparison of hematological parameters at birth and at six weeks of age. Group A: DCC with UCM at 60 seconds. Group B: DCC with UCM at 120 seconds. Group C: Only DCC for 180 seconds. DCC: delayed cord clamping; UCM: umbilical cord milking.

The baseline hemoglobin levels were similar in all three groups, but at six weeks of age, the mean hemoglobin level was significantly higher in Group B (14.41±1.135 g/dL) and Group C (14.21±1.010 g/dL) as compared to Group A (13.31±0.829 g/dL). On post hoc pair-wise comparison, the difference between Group A and Group B was found to be statistically significant (p=0.036), but the comparison between Group A versus Group C (p=0.18) and Group B versus Group C (p=0.44) was non-significant. Similarly, the mean serum ferritin concentration was significantly higher (p=0.003) in Group B (273.41±51.35 ng/mL) and Group C (259.96±41.00 ng/mL) as compared to Group A (242.27±41.13 ng/mL), but on post hoc pairwise comparison, no statistical difference was noted in Group A versus Group B (p=0.13), Group B versus Group C (p=0.12), and Group A versus Group C (p=0.98) (Figure 2, Table 2).

**Table 2:** Comparison of hematological parameters at birth and at six weeks of age among all three groups. DCC: delayed cord clamping; UCM: umbilical cord milking.

Characteristics	Group A (DCC with UCM at 60 seconds) (N=48) (mean±SD)	Group B (DCC with UCM at 120 seconds) (N=49) (mean±SD)	Group C (only DCC for 180 seconds) (N=48) (mean±SD)	p-value	Post hoc pair-wise comparison p-value		
					Group A vs. Group B	Group B vs. Group C	Group A vs. Group C
Hemoglobin at birth (gm%)	16.06±1.20	16.06±1.25	15.56±1.37	0.088	0.78	0.37	0.53
Hemoglobin at six weeks (gm%)	13.31±0.83	14.41±1.13	14.21±1.01	<0.001	0.04	0.44	0.18
Serum ferritin at six weeks (ng/ mL)	242.27±41.13	273.41±51.35	259.96±41.00	0.003	0.13	0.13	0.98

Among the hemodynamic parameters, BP (both systolic and diastolic), HR, and RR of the babies in all groups were comparable at 30 minutes, 24 hours, and 48 hours (Table 3).

**Table 3:** Hemodynamic parameters: BP (systolic and diastolic), HR, and RR of study subjects at 30 minutes, 24 hours, and 48 hours after birth. DCC: delayed cord clamping; UCM: umbilical cord milking; HR: heart rate; RR: respiratory rate; BP: blood pressure.

Vitals at different intervals in postnatal life	Group A (DCC with UCM at 60 seconds) (N=48) (mean±SD)	Group B (DCC with UCM at 120 seconds) (N=49) (mean±SD)	Group C (only DCC for 180 seconds) (N=48) (mean±SD)	p-value
HR (beats/minute)				
30 minutes	133.18±9.8	134.80±7.87	137.30±8.58	0.06
24 hours	137.30±8.58	133.18±9.68	134.80±7.87	0.06
48 hours	134.80±7.87	137.30±8.58	133.18±9.68	0.06
Systolic BP (mmHg)				
30 minutes	56.32±3.27	57.47±2.78	57.47±2.78	0.19
24 hours	56.56±3.43	56.75±3.28	56.98±3.44	0.19
48 hours	57.47±2.78	57.47±2.78	56.75±3.28	0.19
Diastolic BP (mmHg)				
30 minutes	42.72±2.45	42.64±2.34	42.08±1.89	0.30
24 hours	42.08±1.89	42.72±2.45	42.64±2.34	0.30
48 hours	42.64±2.34	42.08±1.89	42.72±2.45	0.30
RR (per minute)				
30 minutes	38.94±6.31	41.44±7.46	41.96±7.46	0.07
24 hours	41.96±7.46	38.94±7.31	41.44±7.46	0.07
48 hours	41.44±7.46	41.96±7.46	38.94±7.31	0.07

At 30 minutes and 24 hours of life, there were no neonatal complications noted in any of the groups. At 48 hours of life, two neonates each in Groups A and B and three neonates in Group C developed NNH, which was not statistically significant. There were no side effects such as RDS, polycythemia, or jitteriness noted.

## Discussion

Umbilical cord management is considered an effective technique to improve arterial oxygen and oxygen delivery to body tissues. DCC and UCM are the main strategies of umbilical cord management that build up the iron stores along with a better hemodynamic state of a newborn. In our study, we found that DCC followed by UCM at 120 seconds is an efficient technique when compared to DCC with UCM at 60 seconds in maintaining the hematological parameters (hemoglobin and serum ferritin) at higher levels in neonates at six weeks after birth. Only DCC for 180 seconds was found to be comparable with the rest of the techniques.

Similar benefits of UCM along with umbilical cord clamping were observed by Alzaree et al. [15]. Improvement in hematocrit at 48 hours of life after UCM was also demonstrated by Mangla et al. [16]. It is a helpful technique, especially in countries with a high incidence of anemia in neonates and children. The benefits of DCC when compared to ECC in near-term infants were also observed by Rashwan et al. [17]. The benefit of the placental transfusion technique on the increase in serum ferritin level is unique to our study.

We also observed that DCC with UCM for 120 seconds and DCC only for 180 seconds are comparable and better than DCC with UCM for 60 seconds. Thus, a duration of greater than 60 seconds is required for optimal benefit while combining the two techniques. Chaudhary et al. [18] conducted a study similar to our study setting and concluded that DCC at 30-60 seconds is an effective and safe intervention in a busy setting to reduce neonatal anemia. Andersson et al. [19] found that DCC helps reduce iron deficiency in term newborns until three to six months of age. In our study, we found that DCC with UCM for 120 seconds and DCC for 180 seconds are beneficial in maintaining serum ferritin at six weeks of age when compared to DCC with UCM for 60 seconds. A combination of UCM with DCC for greater than 60 seconds may be an optimal way of increasing the hemoglobin and stored iron levels in late preterm and term neonates delivered in a busy setting of low- and middle-income countries but inferior to DCC with UCM for 120 seconds and DCC for 180 seconds.

In the study by Alzaree et al. [15], there was no significant change in hemodynamic parameters or clinical adverse effects in the first 24 hours of life in both the DCC and UCM groups. In addition, Jaiswal et al. [4] showed that BP and HR were comparable in both milking and delayed clamping groups and within the normal range in various gestations. Straňák et al. [20] concluded that both UCM and DCC improve hemodynamics in neonates. A similar finding was obtained in our study.

No serious adverse events were observed in all three techniques in our study. A lower proportion of neonatal polycythemia and neonatal jaundice with placental transfusion techniques was also observed by Sura et al. [21].

The strength of our study lies in the robust randomized control design with triple blinding and allocation concealment. It was conducted in a very busy public health facility catering to the poor population of rural areas, which can be of practical use as the majority of deliveries in India happen in similar facilities.

The limitations of our study remain the short duration of follow-up. In addition, in the milking technique, the cord was first cut and then milked, which could have limited the filling of the umbilical cord.

## Conclusions

Both DCC with UCM at 120 seconds and DCC alone for 180 seconds demonstrate equal effectiveness. However, employing DCC followed by UCM at 120 seconds proves to be a more efficient technique compared to DCC with UCM at 60 seconds in maintaining high levels of hematological parameters (hemoglobin and serum ferritin) in neonates at six weeks post-birth. All three techniques exhibit safety concerning adverse events and complications. Future clinical trials with extended study durations can offer more insights into determining the optimal technique among the three for achieving optimal long-term outcomes and addressing potential rare side effects.
